# Validating a common tick survey method: cloth-dragging and line transects

**DOI:** 10.1007/s10493-020-00565-4

**Published:** 2020-11-26

**Authors:** Pia L. Kjellander, Malin Aronsson, Ulrika A. Bergvall, Josep L. Carrasco, Madeleine Christensson, Per-Eric Lindgren, Mikael Åkesson, Petter Kjellander

**Affiliations:** 1 Department of Biomedical and Clinical Sciences, Linköping University, Linköping, Sweden; 2 Grimsö Wildlife Research Station, Department of Ecology, Swedish University of Agricultural Sciences, SLU, Riddarhyttan, Sweden; 3 Department of Zoology, Stockholm University, Stockholm, Sweden; 4 Department of Basic Clinical Practice, University of Barcelona, Barcelona, Spain; 5 Clinical Microbiological Laboratory, Laboratory Medicine, County Hospital Ryhov, Jönköping, Sweden

**Keywords:** *Ixodes*, Repeatability, Tick, Dragging, Total deviation index, Validation

## Abstract

Cloth-dragging is the most widely-used method for collecting and counting ticks, but there are few studies of its reliability. By using cloth-dragging, we applied a replicated line transects survey method, in two areas in Sweden with different *Ixodes ricinus* tick-densities (low at Grimsö and high at Bogesund) to evaluate developmental stage specific repeatability, agreement and precision in estimates of tick abundance. ‘Repeatability’ was expressed as the Intraclass Correlation Coefficient (ICC), ‘agreement’ with the Total Deviation Index (TDI) and ‘precision’ by the coefficient of variation (CV) for a given dragging distance. Repeatability (ICC) and agreement (TDI) were higher for the most abundant instar (nymphs) and in the area of higher abundance. At Bogesund tick counts were higher than at Grimsö and so also repeatability, with *fair* to *substantial* ICC estimates between 0.22 and 0.75, and TDI ranged between 1 and 44.5 counts of difference (thus *high* to *moderate* agreement). At Grimsö, ICC was *poor* to *moderate* and ranged between 0 and 0.59, whereas TDI remained low with estimates lower or equal to 1 count (thus *high* agreement). Despite a 100-fold lower abundance at Grimsö, the same level of precision for nymphs could be achieved with a 70% increase of dragging effort. We conclude that the cloth-dragging technique is useful for surveying ticks’ and primarily to estimate abundance of the nymphal stage, whereas it rarely will be recommended for larvae and adults.

**Electronic supplementary material:**

The online version of this article (10.1007/s10493-020-00565-4) contains supplementary material, which is available to authorized users.

## Introduction

With increased temperature worldwide as climate change progresses, changes in the spatial and temporal distribution of vector-borne diseases among humans and animals are expected and this is likely to have socioeconomic consequences (Campbell-Lendrum et al. 2015; Salman and Estrada-Peña 2013). Ticks (Acari: Ixodidae) are, second to mosquitos, the most important vector of human vector borne diseases (Parola and Raoult 2001). As tick-borne diseases become more common (Salman and Estrada-Peña 2013), reliable surveys of tick abundance and distribution will be needed to implement efficient preventive measures (Slunge 2017). Apart from estimating tick abundance and variation over time (Mejlon 1997), tick surveys have been used to describe prevalence and local variation in tick-borne pathogens (e.g., Kirstein et al. 1997; Jaenson et al. 2009) and to study tick distribution in relation to vegetation and climate change (Lindström and Jaenson 2003).

In Europe, including Sweden, the most common tick and the most important disease vector is *Ixodes ricinus* (Salman and Estrada-Peña 2013). These ticks have three developmental stages: larva, nymph and adult (female and male), where each immature stage requires blood-feeding on a host before molting. In the adult stage, fertilized and fed females lay a single batch of several thousands of eggs, and larvae occur in clusters close to where the eggs hatch (Medlock et al. 2013; Parola and Raoult 2001). With the tick as vector, pathogens may spread from one host to the next (e.g., humans) and larvae therefore rarely act as microorganism transmitters (Salman and Estrada-Peña 2013). The exceptions that exhibit transovarial transmission are *Borrelia* spp*.* and *Rickettsia* spp. (Hauk et al. 2020). To understand and predict tick population dynamics, including spatial and temporal demographic variation and changes in abundance and risk of infection to humans and animals, a reliable and precise estimate of questing (host-seeking) tick abundance is a prerequisite, as proposed in earlier studies (Rynkiewicz and Clay 2014).

The most widely used tick sampling method is cloth-dragging, where an approximately 1-m^2^ large cloth (of, e.g., flannel), is dragged over vegetation. It is considered simple to standardize and easily comparable between studies (Mays et al. 2016). Cloth-dragging for ticks is usually performed as a line transect survey method (LTSM), i.e., with predetermined lines in the study area used for repeated observations or collections to estimate the abundance of a species (Rulison et al. 2013; Schultze et al. 1997). The LTSM is a widely used method in ecological studies for estimating species diversity and abundance of different kinds of organisms (Nomani et al. 2012), including mammals (Plumptre 2000; Romero et al. 2016), birds (Sutherland 2006), marine species (Colin et al. 2013), plants (Kenny et al. 2018) and insects (Kral et al. 2018). Although recommended, validation of the precision and repeatability of LTSM is rarely done (Plumptre 2000) and may be challenging, especially when applied to organisms that move or are displaced after sampling (Colin et al. 2013). In a LTSM study estimating species composition, Kenny et al. (2018) found declining repeatability between repeated surveys when the number of detected species increased, whereas LTSM´s developed for coral reefs were found to be repeatable (Nadon and Stirling 2006).

Studies of relative tick abundance have mainly been evaluated in relation to efficiency and accuracy of different sampling methods (Mays et al. 2016; Rulison et al. 2013), whereas few evaluations of precision and repeatability per se have been done. In this study, we assess repeatability and precision of the cloth-dragging LTSM for the various developmental stages of *I. ricinus* at two sites with different tick densities. More specifically we focus on the following two questions: (1) is sampling with cloth-dragging LTSM repeatable, as proposed in earlier studies (Colin et al. 2013), and (2) what effort is required to generate an abundance estimate with a given confidence, in areas with different environmental settings and thus different tick densities? Repeatability and agreement were evaluated by comparisons of paired samples along the same transect. As ticks are displaced after sampling, we conducted parallel transect lines in order to achieve a minimum estimate of repeatability of cloth-dragging.

## Materials and methods

### Study areas

**Fig. 1:**
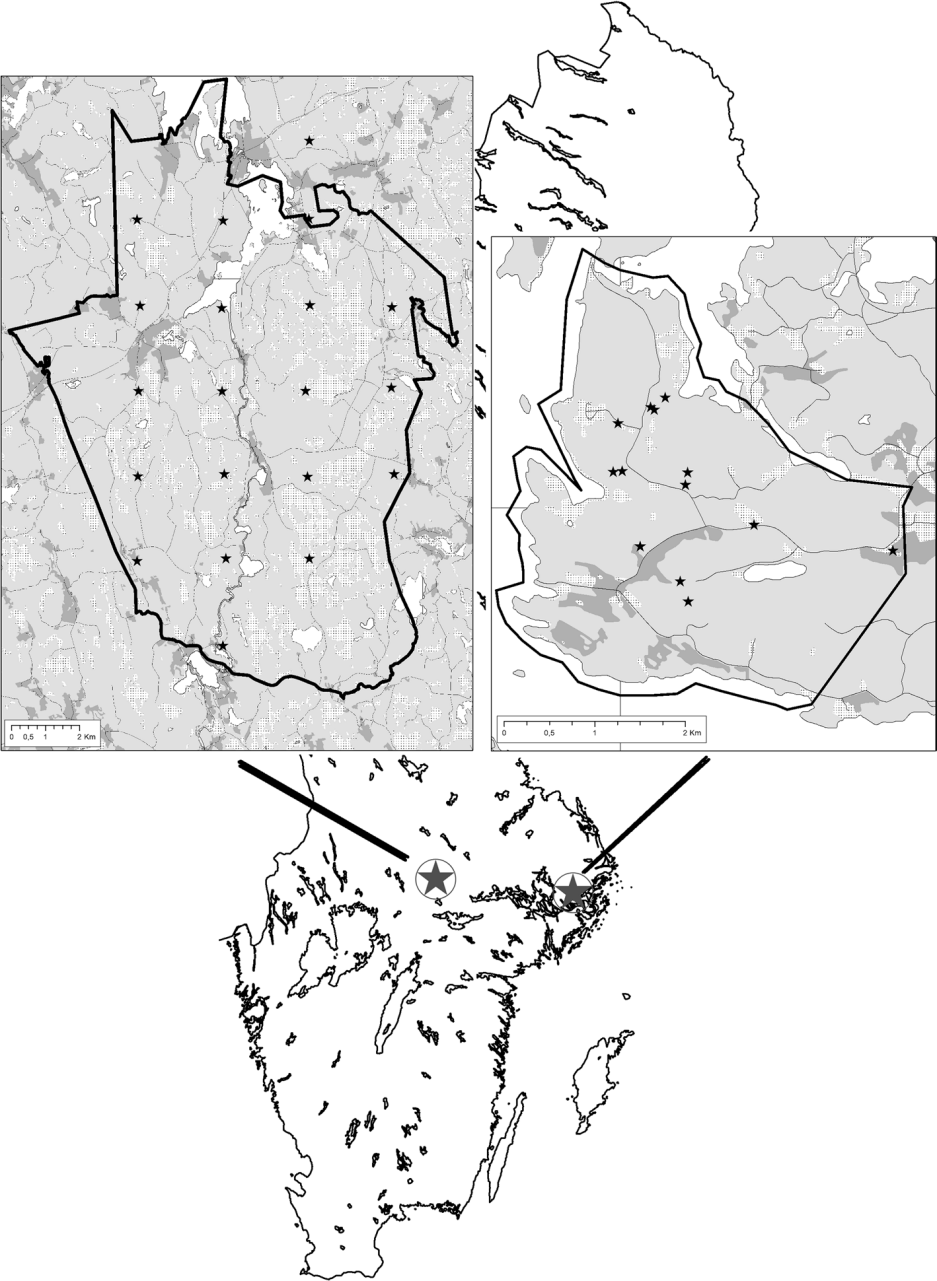
The geographical location of the two study areas Grimsö (low tick density, left panel) and Bogesund (high tick density, right panel). Stars indicate transect locations and differences in areas indicates different habitats (light grey: forest; dark grey: agricultural area; white: water; dotted: bogs and mires). Source: GSD-Terrängkartan, vektor Lantmäteriet

The study was performed in two areas in south central Sweden. Although Grimsö and Bogesund are only 163 km apart (see Fig. 1), they are in different ecotones with different tick-host communities (Davis et al. 2016). Previous fieldwork indicates a much higher tick abundance at Bogesund than at Grimsö, and this observation is in accordance with reports about human cases of tick-borne encephalitis (TBE) from the two areas. Since 2004 TBE is notifiable in Sweden (Public Health Agency of Sweden, www.folkhalsomyndigheten.se; accessed 3 March 2020) and between 2004 and 2018 there were zero human TBE cases from Grimsö (Wallenhammar et al. 2020), whereas Bogesund was marked as a risk area for TBE in 2015 (‘Smittskydd’ Stockholm, www.https://vardgivarguiden.se/kunskapsstod/smittskydd/; accessed 4 June 2020).

Grimsö is a 130-km^2^ area, situated in the boreal zone at the southern edge of the Nordic Taiga, 150 km northwest of Stockholm (59°73′N, 15°47′E). Altitude ranges from 75 to 180 m above sea level and the ground is normally snow covered for about 130 days during December to April. The area consists of mixed coniferous forest (*Picea abies* and *Pinus sylvestris*; 74%), bogs and mires (18%), lakes and rivers (5%) and only 3% agricultural land. The mammal tick-host community is characterized by cyclic populations of rodents (*Apodemus flavicollis*, *Apodemus sylvaticus*, *Clethrionomys glareolus*, *Microtus agrestis*, *Myopus schisticolor*) and also the common shrew (*Sorex araneus*) (Angelstam et al. 1985; Kjellander and Nordström 2003) and other mammals such as moose (*Alces alces*), wild boar (*Sus scrofa*), roe deer (*Capreolus capreolus*), red fox (*Vulpes vulpes*), mountain hare (*Lepus timidus*), European hare (*Lepus europaeus*), lynx (*Lynx lynx*) and wolf (*Canis lupus*) (Nordström et al. 2009). Bogesund is a 12-km^2^ area, situated on a mainland peninsula in the hemiboreal zone 10 km north of Stockholm (59º23′N, 18º15′E), with an altitude from 0 to 60 m a.s.l., normally snow covered for about 80 days during December to March. The area is characterized by a mosaic of mixed forests (*P. abies* and *P. sylvestris* interspersed with *Betula* spp. *Salix* spp., *Querqus robur* and *Tilia cordata*; 65%), agricultural land (25%) and bogs and bare rock (10%). The only human settlements in the area are two farms and a handful of summerhouses. Bogesund has noncyclic rodent populations (*A. flavicollis*, *A. sylvaticus*, *C. glareolus*) and populations of large mammals, including moose, wild boar, roe deer, red fox, mountain and European hare (Angelstam et al. 1985; Kjellander and Nordström 2003). For more detailed descriptions of Grimsö and Bogesund see Davis et al. (2016).

### Sampling

Sampling of questing ticks was performed by dragging a flannel cloth flat to the ground and vegetation (Gray and Lohan 1982). The collected ticks will represent the absolute population in the area; thus, the result will show relative abundance. It is not possible to estimate the absolute abundance with this method. The cloth used was a 1 × 1.35 m white flannel-blanket with the leading edge attached by its short side to a 1.3-m long, wooden dowel, 2 cm in diameter, and a thin rope fastened at each end of the dowel was used to drag it. The end of the cloth was equipped with weights (curtain weights or string chains), to increase contact with the undergrowth. The length of the rope handle (190–250 cm) was adjusted to fit the height of the field worker, so the cloth could be dragged flat to the ground.

Cloth-dragging was performed in 2016, every 2nd week from April 1 until October 31, during daytime after and before the dew and not when it was raining, along 33 line transects in the two study sites (20 and 13 transects in Grimsö and Bogesund, respectively), resulting in a total of 426 dragging occasions (260 and 166 in Grimsö and Bogesund, respectively). The cloth was dragged along transect lines, averaging 90 m (65–110 m, although on six occasions, only 20–50 m of the transects could be dragged, due to flooding). The observer started the cloth-dragging at one end of the transect line and dragged the cloth to the other end (round A), keeping to one side of the line, followed by removal and counting of ticks attached to the cloth. The transect was immediately repeated by the same observer by turning and dragging back to the starting point along the other side of the transect line, parallel to and approximately 1–2 m from round A, resulting in round B, followed by a new tick count. We assume that the rounds were close enough to be done under environmentally similar conditions (e.g., concerning humidity, light, temperature) but far enough to avoid that the tick removal in round A had a direct impact on round B. All ticks were put in vials for later determination of species and developmental stage, using Bristol University Tick ID (www.bristoluniversitytickid.uk) and Becker (2002). All data and codes can be assessed from the Dryad Digital Repository (https://datadryad.org/stash).

To limit the impact of observer identity (n = 23), a single experienced observer trained each person. There are several possible ways in which observer identity may influence both repeatability and average tick count, including individual walking speed during sampling; ways to avoid obstacles (a bush or tree) and the capability to see and search for the sampling objects. With a high number of observers, the heterogeneity is likely increased, and possible influences of observer bias will even out, since the observers are independent of each other, as modelled in Barker et al. (2014).

### Analytical tests

#### Repeatability

As measures of repeatability and agreement we used the Intra-class Correlation Coefficient (ICC) and Total Deviation Index (TDI). The ICC assesses distinguishability (here called *repeatability*), that involves both within-subject(transect)-variance (WSV) and between-subjects(transects)-variance (BSV) and expresses the proportion of the total variation that is reproducible among repeated measurements of the same subject (transect). Thus, the ratio of variation among subjects (BSV) and the total variation (BSV + WSV), here: ICC = BSV/(BSV + WSV) resulting in a dimensionless quantity with a value between 0 and 1 (Shrout and Fleiss 1979). TDI evaluates *agreement* between rounds and only involves WSV. TDI is an unscaled index of agreement of what extent the tick counts from round A and B may differ, thus WSV (Lin 2000) and expressed in the same unit as the analyzed variable, here number of ticks in relation to the mean (N – µ). We use both indices as ICC is highly dependent on BSV whereas TDI is not. This means that a low ICC can be observed because of low BSV even in case the agreement was high (Carrasco et al. 2014). The TDI estimates will help to evaluate the extent of agreement and to indicate a potential masking of the ICC due to low BSV.

Point estimate and 95% confidence interval (95% CI) for the ICC of tick counts of each round was estimated, using a general linear mixed model (GLMM) as proposed in Carrasco (2010) and with ‘transect ID’ as a random effect and ‘round’ as a fixed effect, using the glmmTMB package (Brooks et al. 2017) in R v.4.0.0 (R Core team 2017). A first model with log link, Poisson error distribution and ‘transect’ as normally distributed random effect was fitted. Overdispersion was checked by means of half-normal plots (Demetrio et al. 2014). In cases of overdispersion a negative binomial error distribution with log link was fitted.

At Grimsö very few larvae or adults were found, and many transects had 0 counts of these three groups (larvae: 253, males: 244, females: 251, out of 260 transects; additional data are given in Suppl. File 1–4). This made the use of statistical distributions and GLMM’s unsuitable. Instead, a non-parametric estimate of the ICC was obtained for these three developmental stages, by means of the weighted Kappa index using Fleiss-Cohen (quadratic) weights (Lin et al. 2007).

The strength of repeatability was classified in accordance with the scale proposed by Landis and Koch (1977) in the agreement setting, where a value < 0 is *poor*, 0–0.20 is *slight*, 0.21–0.40 is *fair*, 0.41–0.60 is *moderate*, 0.61–0.80 is *substantial* and 0.81–1.0 is (*almost*) *perfect.*

Additionally, we present TDI_90%_, as the difference in tick counts between rounds A and B that can be reached with 90% probability, thus the maximum absolute difference between round counts at the same transect with a probability of 90%. One-tailed 95% CI was estimated by bootstrapping (Perez-Jaume and Carrasco 2015), thus presenting the 95% confidence upper bound (UB).

To set an overall quantitative definition of TDI_90%_ in terms of *low* to *high* agreement between rounds A and B is not straightforward because TDI_90%_ is expressed as an absolute mean number. In order to propose a scale to interpret the TDI values we used a wide definition and based that on human behavior and risk perceptions in relation to their protective behavior against tick bites and related diseases (Slunge et al. 2019) in combination with tick expert opinions, as in former studies using TDI (Balaguer et al. 2016). Slunge et al. (2019) conclude that only 48% of the respondents in a study about risk perceptions, avoid certain high tick-risk habitats and even fewer respondents (43%) consider ticks as a serious health risk. Thus, even a rough estimate of tick abundance will be good enough for most purposes—currently, the societal need to prevent and inform about or to pay for a higher precision seems not to be motivated. Therefore, considering prior knowledge of different developmental stages, a 4-level qualitative classification of TDI_90%_ was determined (*low*, *moderate*, *good*, *high*), a truncation suitable for larvae and all developmental stages combined was used (> 20, 10–20, 5–10, < 5) and another for nymphs and adults (> 9, 6–9, 3–6, < 3).

The Wald test (Sokal and Rohlf 1995) was used to test for systematic difference between rounds A and B, by using round parameter estimate of the fitted GLMM.

#### Sampling effort

**Fig. 2:**
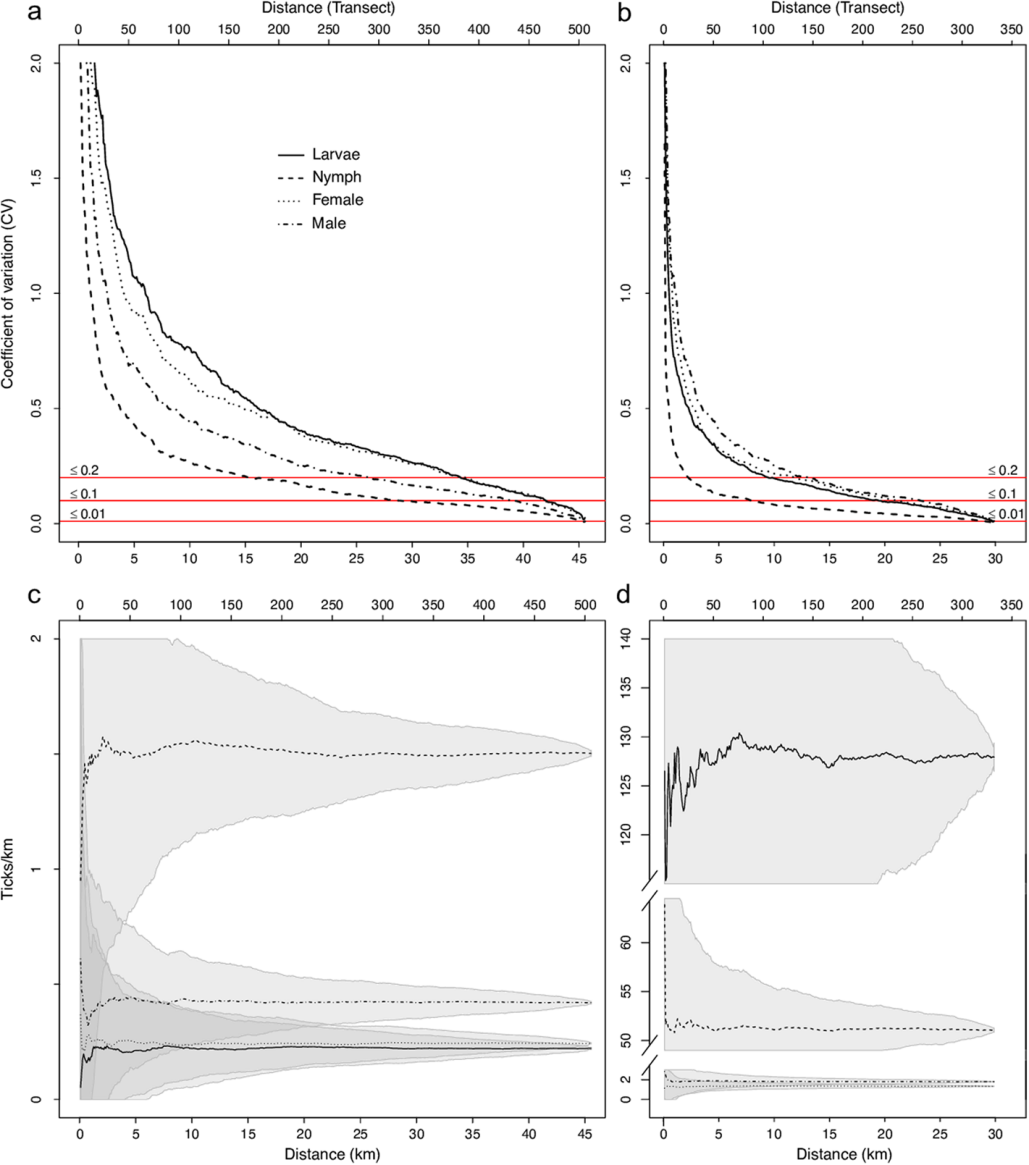
The coefficient of variation (CV) in tick counts after permutation of single transects in Grimsö (n = 506; **a**) and in Bogesund (n = 332; **b**), CV = 0.01, 0.1 and 0.2 highlighted horizontal lines, to illustrate the dragging distance (in km) required to reach the preset target variances, and mean number of ticks km^−1^ sampled at Grimsö (**c**) and Bogesund (**d**), where shaded areas illustrate the standard deviation (SD) at different dragging distances. Notice that in each panel the bottom x-axis represents distance (km) and the top x-axis transects. The scales for mean number of ticks km^−1^ differ between the two areas with a discontinuous y-axis in **d**. (For complete CV-polygons, see additional data in Suppl. File 5)

Sampling effort was defined as the dragging distance required to estimate relative tick abundance with a given confidence and was estimated using accumulation curves (Krebs 1999). Longer dragging distance results in more ticks sampled and assuming that tick density is constant across transects in the same area, the confidence in the mean number of ticks per distance unit, is expected to increase. In this study, coefficients of variance (CV = standard error/mean) lower than 0.01, 0.1 and 0.2 were used as cut-off values, to compare differences in abundances between the two study areas and among developmental stages. A pre-defined target value of variance can only be decided in relation to the aim of the survey. Consequently, the target value may need to be adjusted and we therefore present the data in a way that enables the evaluation of different targets (Fig. 2a, b), made in R (R Core team 2017). For this analysis, we used rounds A and B from each transect as separate dragging rounds. To exclude unwanted effects of seasonal impact we used permutations to randomize the survey date (occasion). After adding a one-way round to the calculation, a new randomization was performed, so a new one-way round was added to the previous, thus in total comprising 506 and 332 randomizations for Grimsö and Bogesund, respectively.

## Results

In total, 5569 ticks were sampled from 75 km of cloth-dragging of 33 line transects. The mean number of ticks per km was 2.37 (95% CI = 1.82–3.09) at Grimsö and 230 (95% CI = 160–331) at Bogesund (Table 1). Mean number of ticks per transect for the separate developmental stages varied from 0.02 (95% CI = 0.01–0.04) for larvae and females up to 0.15 (95% CI = 0.10–0.23) for nymphs at Grimsö (Table 1), and from 0.13 (95% CI = 0.09–0.19) for females up to 11.5 (95% CI = 8.65–15.3) for larvae at Bogesund (Table 1).

**Table 1:**
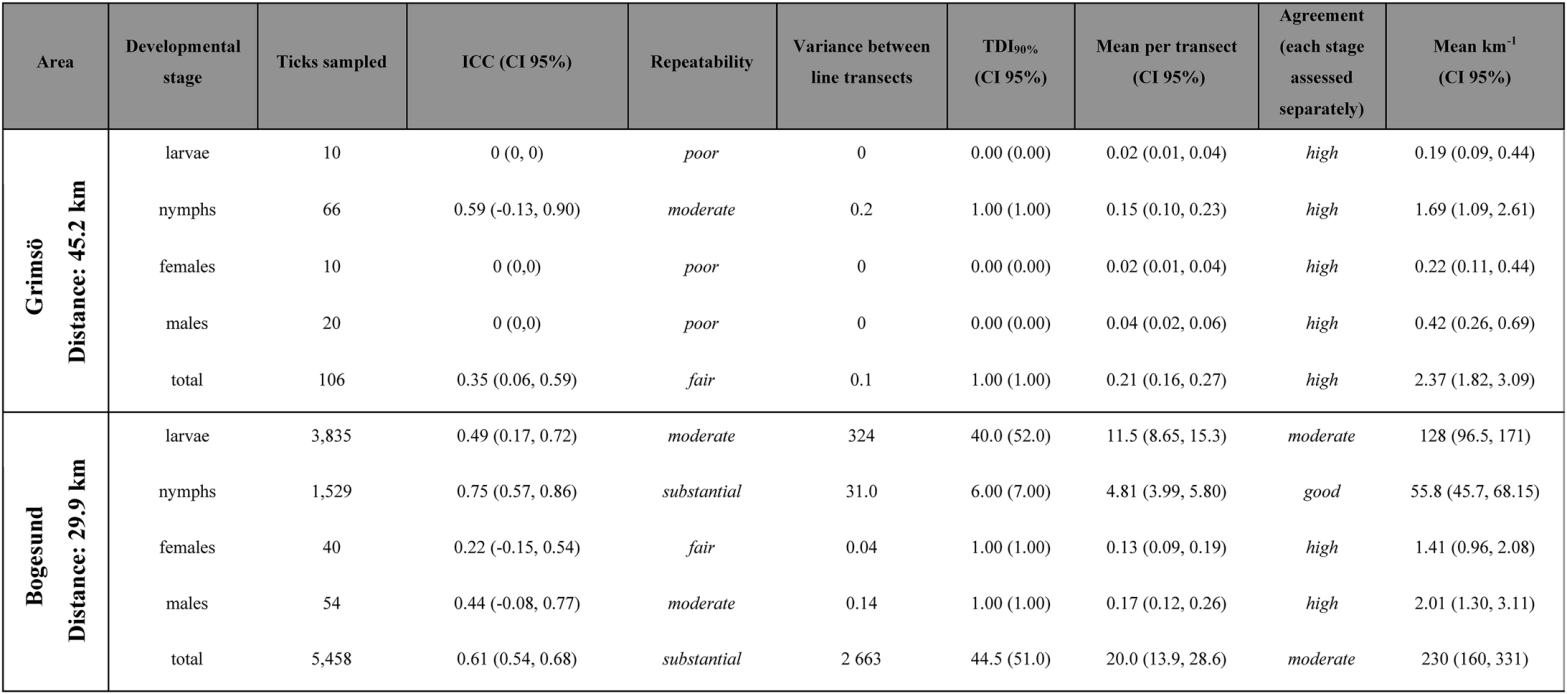
Number of ticks sampled for each developmental stage, repeatability (ICC) and agreement (TDI_90%_, UB), between rounds A and B, together with the strength classification (*poor*, *slight*, *fair*, *moderate*, *substantial*, and *perfect*) for ICC from Landis and Koch (1977) and the 4-leveled classification for TDI_90%_ (*low*, *moderate*, *good* and *high*), mean tick counts per transect and km and the variance in tick counts between line transects. In the right column the estimated tick abundance (mean no. km^–1^) is given. Ticks are collected in two study areas, Grimsö and Bogesund in south-central Sweden during 2016

No systematic differences were found between rounds A and B for any development-stage or area where, 95% CIs for the ratio of means (for rounds A and B) included the value of 1 in all cases.

### Repeatability

At Grimsö, the ICC between rounds A and B with all developmental stages pooled: total was *fair* (mean ICC = 0.35, 95% CI = 0.06–0.59; Table 1). However, when analyzing each developmental stage separately the ICC was *poor* for all apart from nymphs, for which ICC was *moderate* (0.59, 95% CI = −0.13 to 0.90). The agreement (TDI_90%_) was 0 at Grimsö for all developmental stages, apart from nymphs where it was 1.0 (i.e., for nymphs, the maximum difference between round A and B counts at the same transect is 1.0). All developmental stages were assessed separately, but all showed *high* agreement between rounds within the same transect (Table 1).

At Bogesund, the ICC with all developmental stages pooled: total was *substantial,* (mean ICC = 0.61; 95% CI = 0.54–0.68; Table 1) thus higher than at Grimsö. Among the separate developmental stages, repeatability varied between *fair* for females (mean ICC = 0.22; 95% CI = −0.15 to 0.54) and *substantial* for nymphs (mean ICC = 0.75; 95% CI = 0.57–0.86; Table 1). Assessed separately, the TDI_90%_ at Bogesund could be considered *high* for females and males, *good* for nymphs and *moderate* for larvae and total count (Table 1).

### Sampling effort

To reach CV ≤ 0.1, in the overall mean of total number of ticks per km at Grimsö, would require 26.5 km of dragging. It would require, 42.8 km for the larval stage, 31.3 km for nymphs, 41.5 km for females and 39.2 km for males, to achieve the same precision for developmental stages separately (Fig. 2a, c, Suppl. File 5a). At Bogesund on the other hand, 15.4 km of dragging was enough to reach the same targeted precision (CV ≤ 0.1) for the overall estimate of mean tick abundance, and 20.0 km (larvae), 7.9 km (nymphs), 21.7 km (females) and 23.0 km (males) for the various developmental stages (Fig. 2b, d, Suppl. File 5b). To compare the total (pooled) number of ticks in the two areas with the same precision, an increased effort of almost 70% (from a 15.4 km dragging distance at Bogesund to 26.5 km at Grimsö) is needed. Alternatively expressed, after dragging for example 20 km, a precision of CV = 0.14 and 0.07 is reached at Grimsö and Bogesund, respectively. In this example, for CV ≤ 0.2, 11.4 and 6.6 km, and for CV ≤ 0.01, 45.3 and 29.6 km is required at Grimsö and Bogesund, respectively, for the total number of ticks. Finally, at Grimsö with a low mean number of total counted ticks of 2.37 the 95% CI was narrow (1.82–3.09), in contrast to Bogesund with a high mean number of total counted ticks of 230 and 95% CI = 160–331 (Table 1).

## Discussion

We assessed the agreement and repeatability of relative abundance estimates made of questing ticks belonging to different developmental stages of *I. ricinus*, using a LTSM form of cloth-dragging method and evaluated the effort needed to achieve accurate estimates. We did this by comparing tick counts between transects and along parallel drags of the same transect in two areas (Grimsö and Bogesund) with different tick densities throughout the tick activity season.

### Repeatability and agreement

The usefulness of a combination of repeatability (ICC) and agreement (TDI) to understand the accuracy of a method (Carrasco et al. 2014) is clearly illustrated from this study with tick developmental stages and areas that differ in tick abundance and incidence of 0 counts. With TDI, the agreement between rounds A and B was often *high*, especially when the number of sampled ticks was low. This is induced by the fact that TDI only takes WSV and the numerical difference between rounds A and B into account. Counts of larvae at Grimsö and adults in both study sites were rarely above 1 and the majority of transects were 0 (additional data are given in Suppl. File 1–4). Thus, the numerical difference between rounds A and B can only reach one count (tick) with a probability of 90% (Lin 2000). A statistical consequence gives that high absolute mean values will more easily result in *low* agreement between rounds A and B (as in larvae at Bogesund) while still being *good* for nymphs.

Even though repeatability (ICC) positively depends on agreement between rounds A and B, it also carries information about the ability to discriminate one transect from another (Carrasco et al. 2014). Contrary to the agreement, repeatability tended to be lower for absolute means that had lower counts. This is thus not explained by *poor* agreement but rather by the relatively low variation in tick counts between transects. The developmental stage with the highest ICC was nymphs, that was *moderate* at Grimsö and *substantial* at Bogesund (Table 1). As the agreement at Grimsö was larger than at Bogesund, the lower repeatability at Grimsö is more likely due to a relatively lower variation between transects in this site compared to Bogesund, as even small disagreements (e.g., one tick difference between rounds A and B) has a strong negative impact on ICC.

Ticks are small animals and live their lives within an area of a couple of square meters if not transported further by a host (Salman and Estrada-Peña 2013), so some differences in number of ticks between transects were expected. Environmental factors like time of day, season, weather, vegetation and microclimate may affect tick abundance and questing activities, hence creating differences between transects (Salman and Estrada-Peña 2013). Here we constrained these environmental effects by estimating agreement and repeatability from paired rounds of cloth-dragging of each transect at closely spaced time intervals. However, the LTSM in our study is a destructive survey in the sense that objects (ticks) were removed from transects by cloth-dragging. We were thus unable to repeat the survey on exactly the same transect line, but rather on a line 1 m away and parallel to the first round. With this procedure we constrain many sources of environmental variation except those that act on the scale of the distance between the two rounds, e.g., differences in microclimate and vegetation structure. No systematic difference in average tick counts could be found between rounds A and B, indicating that the tick-removal in round A had no or at least limited impact on round B.

The large difference in larvae counts both in relation to the other developmental stages and between the two areas, may be explained by a heterogeneous distribution of synchronously hatched larvae from eggs laid in one place whereas individuals of the other developmental stages are expected to be distributed more evenly in the landscape (Medlock et al. 2013). The chance of dragging through a cluster of hatched larvae is likely to be low in areas with low tick abundance as at Grimsö. In areas with generally higher tick abundance as Bogesund, there is a higher chance to collect larvae. Consequently, tick abundance and distribution in combination will strongly influence repeatability (Medlock et al. 2013; Nomani et al. 2012).

After assessing agreement and repeatability of the cloth-dragging method using LTSM, it appears to be reliable in both low- and high-density areas to estimate approximate tick abundance of the most common developmental stage (nymphs) and overall tick abundance, but is less reliable to estimate the abundance of larvae, females and males.

### Sampling effort

We found that the dragging effort required to reach a given pre-defined target precision (CV) increased with lower tick abundances. Mean number of ticks (stages pooled) were approximately 100× higher at Bogesund compared to Grimsö (Table 1) and approximately a 70% increased effort was needed at Grimsö to reach the same precision as at Bogesund. To detect a relative proportional change of abundance it would thus require a much higher effort at Grimsö than at Bogesund. Among the various developmental stages, nymphs required the least effort in both areas and reached a stable mean (when CV ≤ 0.1) after 31.3 km at Grimsö compared to 7.9 km at Bogesund. Adults that normally represent the less abundant developmental stages (Kirsten et al. 1997), as at Bogesund, required a higher dragging effort than nymphs. At Grimsö, larvae were even less abundant and required the highest effort, due to a very low abundance with many zeroes. The mean count of adults was low in both areas and the differences were approximately 1 km^−1^ for both females and males, but that is enough to require twice the effort at Grimsö (approx. 40 km) compared to Bogesund (approx. 20 km), to generate the same precision (CV).

At Bogesund, larvae—the most common developmental stage—required a longer dragging distance to stabilize the CV compared to nymphs, something that may be explained by the clustered distribution of larvae (Medlock et al. 2013). Larvae counts were almost 700× higher at Bogesund (128 km^−1^; 95% CI = 96.5–171) compared to Grimsö (0.19 km^−1^; 95% CI = 0.09–0.44) and needed less effort to reach the same precision as at Grimsö (Fig. 2, Suppl. File 5).

To make the best of a limited budget, effort and precision should ideally be pre-defined in relation to the aim of the study. The required precision for a population survey depends on how it will be used. For example, a tick survey with the aim to support preventive health care presents differences in tick densities between areas (Slunge 2017). Thus, yearly or seasonal estimates will most likely benefit from an equal precision. This applies particularly when comparing high-density areas, as an increase in abundance of for example 20% would mean a proportionally greater number of tick bites (e.g., Bogesund where 56 nymphs per km would increase to 67 ± 7, when CV = 0.1); in a low-density-area an increase of 20% would not result in a much greater number of tick bites (e.g., Grimsö where 1.7 ticks per km would increase to 2 ± 0.2). In that case a low precision in low-density-areas can be more acceptable. On the other hand, from an ecological perspective, e.g., with the aim to investigate tick population dynamics, precision could be as important in low-density areas, to follow variation between cohorts and changes in tick demography between years. It is thus usually helpful to decide prior to the start of fieldwork what level of measurement precision a study requires (Krebs 1999). A preset precision—decided by authorities, management or the research question at hand—simplifies a flexible and adaptive method that estimates when the dragging effort is enough.

### Future improvements and recommendations

In this study two areas with contrasting tick densities were used. Including more sites would have been desirable to investigate further effects of environmental variation on the repeatability. Still, with a given budget there is a trade-off between high-intensity sampling at a few sites versus lower-intensity sampling at many sites. The two sites (Grimsö and Bogesund) represent, respectively, the boreal and hemi-boreal forest ecosystems that dominate much of the land area of Fennoscandia (Ingelög 1988).

Dragging long transects as we did may increase tick drop off rate (Li and Dunley 1998) and most likely affect both repeatability and agreement negatively as well as the fact that we dragged rounds A and B in opposite directions. Even though we were unable to measure how these two shortcomings affected our results, it is not a problem per see, as we analyze whether the cloth-dragging method is good enough (repeatability and agreement), given such unknown methodological weaknesses. Thus, all estimates presented in this study of repeatability should be considered as conservative and would most likely improve had it been possible to correct for these and other ‘unknowns’. Still, we raise the caution that the comparability of transects conducted under different conditions will be affected by different rates of drop-off and we call for more studies on this issue.

Despite the many advantages of cloth-dragging LTSM to estimate relative tick abundance, it still does not provide information of absolute tick density. There have been attempts to estimate absolute tick density by capture-mark-recapture studies and removal sampling methods. Daniels et al. (2000) estimated that approx. 6.3% among all developmental stages was obtained in a single sample. Still, validated methods for assessing absolute density for *I. ricinus* needs to be developed in future studies.

## Conclusions

Due to current climate changes, followed by an expected increase of pathogen prevalence in ticks, preventive health care and recommendations must be supported by reliable surveys. We can conclude that (1) the cloth-dragging LTSM is *substantially* repeatable with an ICC of 0.61 (95% CI = 0.54–0.68) for total tick counts and 0.75 (95% CI = 0.57–0.86) for nymphs, and *good* agreement for nymphs (TDI_90%_ 6.0, 95% confidence UB 7.0) in the high-abundance area, and (2) in the low-abundance area increased effort was required to generate a comparable CV for the nymphal tick stage.

We recommend more studies to conduct repeated line transects because (1) replicated measures can be used to increase the accuracy of the estimates, and (2) repeatability can be used to better understand the variation within transects, and thus sets the upper limit of the amount of variation that can be explained by extrinsic factors on estimated tick abundance. Finally, we recommend an adaptive use of estimates of repeatability and effort by accumulation curves in any survey using LTSM.

## Electronic supplementary material

Below is the link to the electronic supplementary material.

Electronic supplementary material 1 (TIF 3966 kb). Supplementary Files 1–4. Contingency tables for all developmental stages of ticks collected in the low-density Grimsö site; larvae (a), nymphs (b), females (c) and males (d), and in the high-density Bogesund site: larvae (e), nymphs (f), females (g) and males (h). The vertical table axis represents data from round A and the horizontal axis represents data from round B. Data were collected in 2016

Electronic supplementary material 2 (TIF 4384 kb)

Electronic supplementary material 3 (TIF 4026 kb)

Electronic supplementary material 4 (TIF 3668 kb)

Electronic supplementary material 5 (TIF 556 kb). Supplementary File 5. The mean number of ticks km^–1^ (solid line) and coefficient of variation (dashed curve) for all developmental stages separately at Grimsö (a) and Bogesund (b). Shaded areas illustrate standard deviation (SD) around the mean. The vertical red lines illustrate the required dragging effort to reach the example of the preset target variance (CV) of ≤ 0.2 (dashed), ≤ 0.1 (solid) and ≤0.01 (dotted, only included for development stages and study areas where this was reached). The scales for mean number of ticks km^–1^ differ between the areas and developmental stages

## Data Availability

Dryad Digital Repository (https://datadryad.org/stash).

## Code availability

Dryad Digital Repository (https://datadryad.org/stash).
