# Supplementary figures and images for: Validating a common tick survey method: cloth-dragging and line transects
Source: Exp Appl Acarol. 2020 Nov 26;83(1):131–46. doi: 10.1007/s10493-020-00565-4 (PMC7736024; doi:10.1007/s10493-020-00565-4)

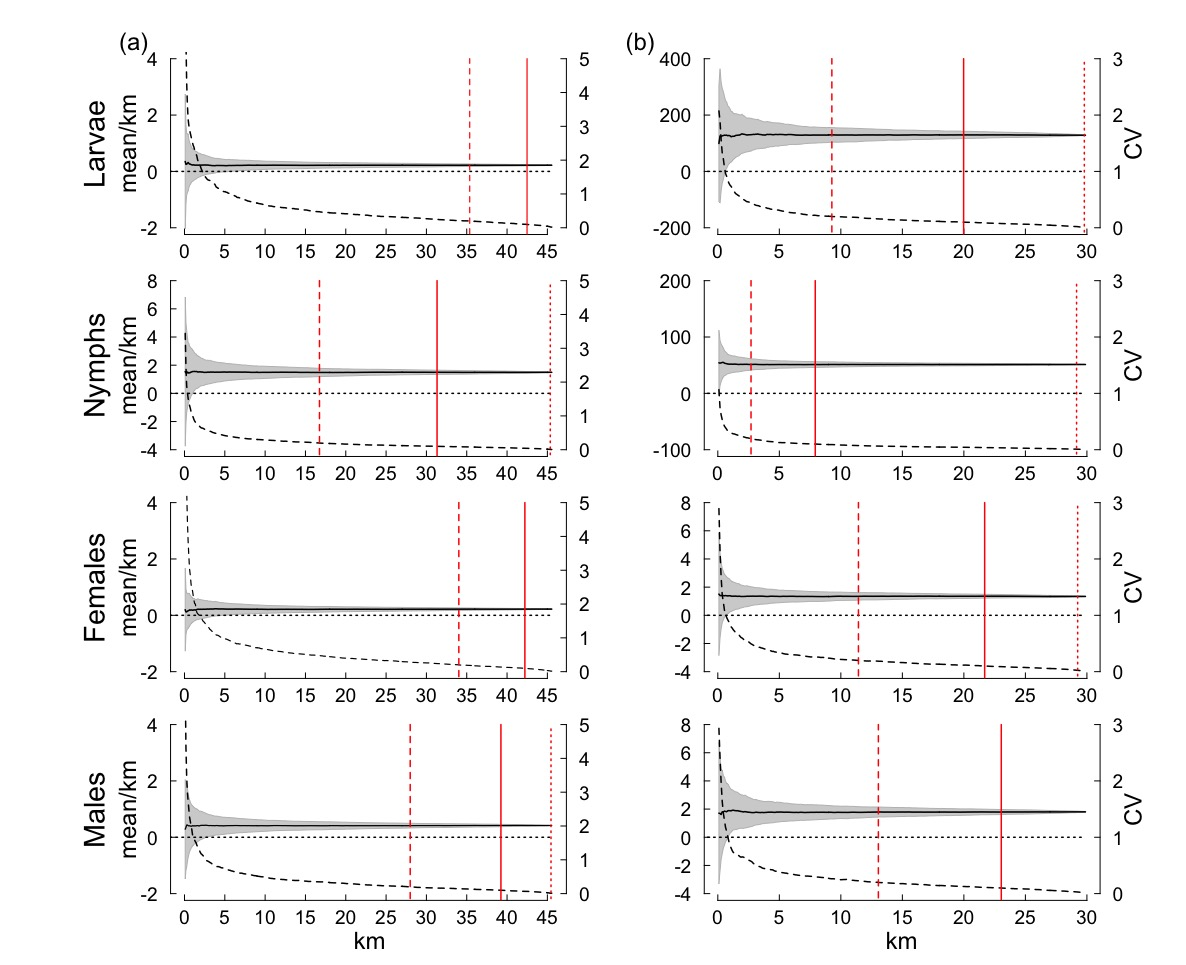

Supplement: Supplementary file 5 — Electronic supplementary material 5 (TIF 556 kb). Supplementary File 5. The mean number of ticks km–1 (solid line) and coefficient of variation (dashed curve) for all developmental stages separately at Grimsö (a) and Bogesund (b). Shaded areas illustrate standard deviation (SD) around the mean. The vertical red lines illustrate the required dragging effort to reach the example of the preset target variance (CV) of ≤ 0.2 (dashed), ≤ 0.1 (solid) and ≤0.01 (dotted, only included for development stages and study areas where this was reached). The scales for mean number of ticks km–1 differ between the areas and developmental stages [file 10493_2020_565_MOESM5_ESM.tif]
